# Tri-Ponderal Mass Index as a Screening Tool for Identifying Body Fat and Cardiovascular Risk Factors in Children and Adolescents: A Systematic Review

**DOI:** 10.3389/fendo.2021.694681

**Published:** 2021-10-21

**Authors:** Jiahong Sun, Rong Yang, Min Zhao, Pascal Bovet, Bo Xi

**Affiliations:** ^1^ Department of Epidemiology, School of Public Health, Cheeloo College of Medicine, Shandong University, Jinan, China; ^2^ Department of Nutrition and Food Hygiene, School of Public Health, Cheeloo College of Medicine, Shandong University, Jinan, China; ^3^ Center for Primary Care and Public Health (Unisanté), University of Lausanne, Lausanne, Switzerland

**Keywords:** children, tri-ponderal mass index, obesity, body fat, cardiovascular risk factors

## Abstract

Because of the limitation of body mass index (BMI) in distinguishing adipose mass from muscle, the tri-ponderal mass index (TMI) has been proposed as a new indicator for better assessing adiposity in children and adolescents. However, it remains unclear whether TMI performs better than BMI or other adiposity indices in predicting obesity status in childhood and obesity-related cardiovascular risk factors (CVRFs) in childhood or adulthood. We searched PubMed, Cochrane Library, and Web of Science for eligible publications until June 15, 2021. A total of 32 eligible studies were included in this systematic review. We found that TMI had a similar or better ability to predict body fat among children and adolescents than BMI. However, most of the included studies suggested that TMI was similar to BMI in identifying metabolic syndrome although TMI was suggested to be a useful tool when used in combination with other indicators (e.g., BMI and waist circumference). In addition, limited evidence showed that TMI did not perform better than BMI for identifying specific CVRFs, including insulin resistance, high blood pressure, dyslipidemia, and inflammation in children and adolescents, as well as CVRFs in adults.

**Systematic Review Registration:**

https://www.crd.york.ac.uk/prospero, CRD42021260356.

## Introduction

The age-standardized prevalence of obesity among children and adolescents aged 5 to 19 years has globally increased from 0.7% in 1975 to 5.6% in 2016 among girls and 0.9% to 7.8% among boys (1). Obesity in children is a cause of several detrimental health outcomes in childhood and later in adulthood, such as left ventricular hypertrophy (2), increased carotid intima-media thickness (3), kidney disease (4), and liver disease (5), cancer, cardiovascular diseases, and death (6–8). Thus, an early and accurate diagnosis of obesity in children and adolescents is urgently needed, in order to reduce the short-term and long-term burden of pediatric obesity-related health outcomes.

Body mass index (BMI, kg/m^2^) is the most widely used physical indicator of adiposity in both children (with overweight/obesity cutoffs based on age and sex percentiles) and among adults (overweight: BMI 25–29; obesity BMI ≥ 30). Although BMI is strongly correlated with adiposity, the indicator cannot distinguish well between excess weight due to increased fat mass or increased muscle mass (9), especially for changes in body composition during adolescence, leading to weight increase being out of proportion of the change in height squared (10, 11). Although the percentage of body fat is suggested as an accurate method for identifying obesity in children and adolescents, it is less applicable for routine health care, as well as in school-based settings (12).

Tri-ponderal mass index (TMI), calculated as weight (kg)/height (m^3^), is an emerging indicator, which has been suggested to predict percent body fat (10) and metabolic syndrome (MetS) (13) at least as well as, or better than BMI. However, findings in other previous studies were inconsistent (14–17). For instance, the prevalence of overweight and obesity was higher when identified with BMI (based on standard deviation score, SDS) than with TMI in children and adolescents aged 6–17 years (14). It was also found that BMI (or BMI z-score or BMI-SDS) predicted MetS better than TMI among adolescents aged 10–17 years (15, 16).

It is however unclear whether the emerging TMI can better identify adiposity in childhood or adolescence than the commonly used BMI (10, 14, 18–26) and better predict obesity-related cardiovascular risk factors (CVRFs) such as high blood pressure, dyslipidemia, insulin resistance, and the MetS in childhood (13–16, 18, 21, 26–34) or adulthood (35–37). The misclassification of obesity may lead to either omissions of children who are at high risk of obesity-related diseases or excessive anxiety due to overdiagnosis and then unnecessary waste of medical resources (38). Identifying potential adiposity-related indicators that can accurately predict body fat or related risks has significant implications for prevention, treatment, and management of pediatric obesity.

Therefore, in order to assess whether TMI can be a substitute for BMI in routine pediatric clinical practice to estimate obesity and related CVRFs in children and adolescents or adults, we reviewed articles on the ability of TMI to identify increased body fat, in children and adolescents, and to predict CVRFs in both childhood and adulthood.

## Methods

### Search Strategy

This review was performed according to the recommendation from the Preferred Reporting Items for Systematic Reviews and Meta-Analyses statement (PRISMA) (http://www.prisma-statement.org/). We searched relevant articles in PubMed, Cochrane Library, and Web of Science until June 15, 2021 using the following search strategy: (“Triponderal mass index” OR “Tri-ponderal mass index” OR “Tri-ponderal index”) AND (“children” OR “childhood” OR “adolescents” OR “adolescence” OR “teenagers” OR “youth” OR “students”) AND (“body mass index” OR “obesity” OR “body fat” OR “cardiovascular disease risk” OR “hypertension” OR “dyslipidemia” OR “insulin resistance” OR “impaired glucose” OR “metabolic syndrome” OR “MetS” OR “inflammation”). We also identified eligible papers from the lists of references in the identified papers. We have registered on PROSPERO (available at: https://www.crd.york.ac.uk/prospero/#aboutpage), and the ID is CRD42021260356.

### Inclusion Criteria and Exclusion Criteria

Inclusion criteria were as follows: 1) original article; 2) body fat or adiposity assessed using TMI and BMI in childhood or adolescence; 3) the paper described the association of TMI and BMI measured in childhood and adult with selected CVRFs [i.e., hypertension; dyslipidemia; insulin resistance (IR) or impaired glucose; MetS; and inflammation] measured either in childhood (e.g., at the same time of measurement of the BMI/TMI, e.g., in cross-sectional surveys) or in adulthood (e.g., cohort studies) or both; and 4) cross-sectional, cohort, or retrospective studies. Exclusion criteria were as follows: 1) obviously irrelevant articles; 2) TMI measured in adulthood; 3) other languages rather than English; 4) letter or comment; and 5) studies without data of interest.

### Identification of Relevant Studies and Data Extraction

Two independent authors (JS and RY) performed the literature search and extracted the data. In case of disagreement between the two authors, a third expert (BX) was consulted to reach an agreement. The information on the first author, publication year, country of origin, study design, sample size, age and sex distribution of the study population, exposures, outcome definition, adjusted covariates, and results was extracted from each eligible study.

### Study Quality Assessment

An 11-item checklist of the cross-sectional study evaluation scale recommended by the Agency for Healthcare Research and Quality (AHRQ) was used to evaluate the quality of cross-sectional studies (39), with answers coded as “Yes” (1) or “No or not clear” (0). The total score of the scale is 11 points. A score of 8 to 11 points is rated as high quality, 4 to 7 points as moderate quality, and less than 4 points as low quality. The Newcastle–Ottawa Scale (NOS) star system (range 0 to 9 stars) was used to evaluate the quality of cohort and case–control studies (40). In brief, four items related to the selection of participants, two items to the comparability of participants, and three items to the exposures or outcomes. A score of “0–3” was regarded as low quality, “4–6” as moderate quality, and “7–9” as high quality.

## Results

### Study Selection

**Figure 1:**
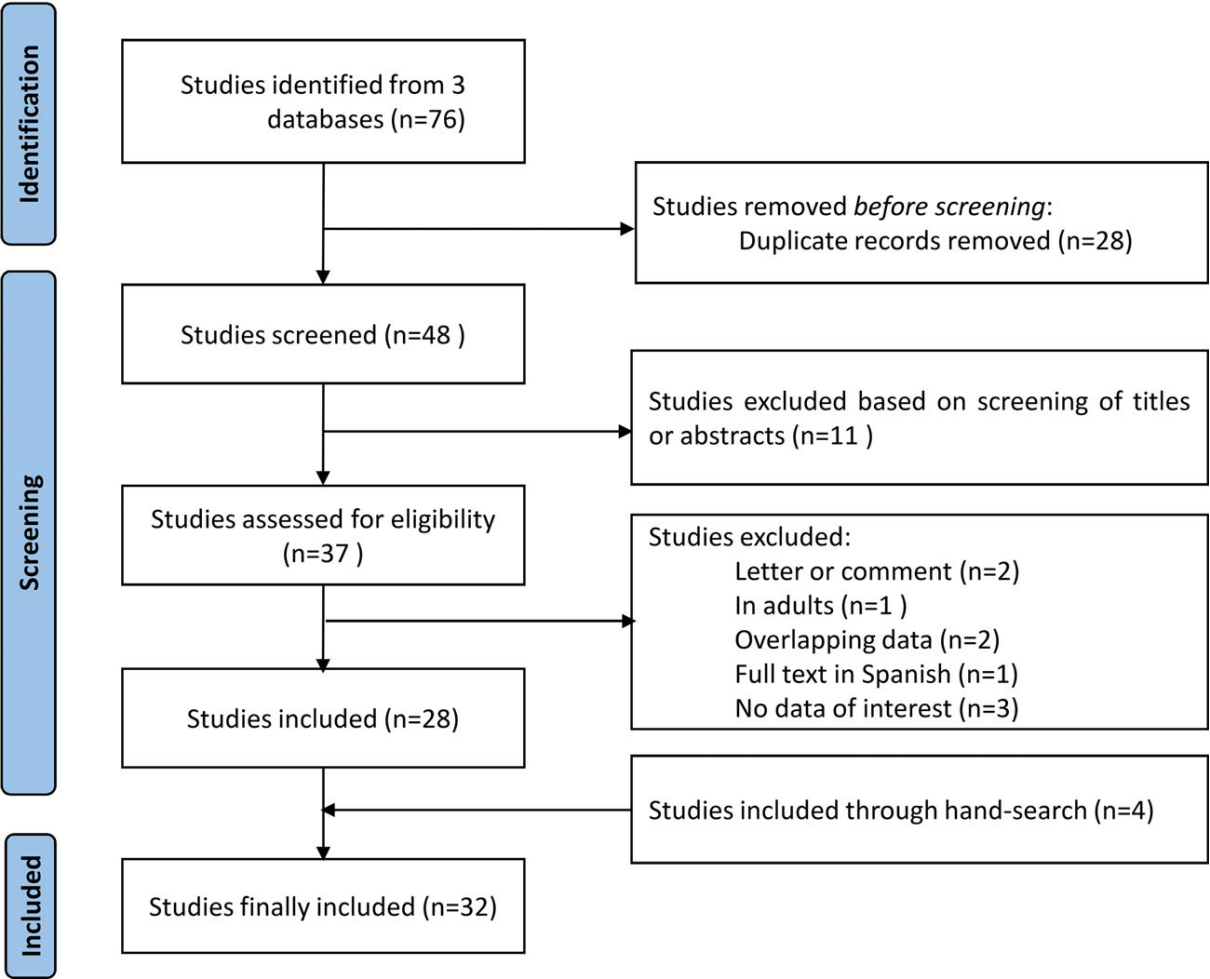
PRISMA flowchart of study selection.

A total of 76 articles were initially identified. After excluding 28 duplicate articles, 48 remained for screening. After excluding 11 irrelevant articles, 2 letters/editorials, 1 in adults, 1 with overlapping data, 1 in Spanish, and 3 with no data of interest, 28 relevant studies were included. In addition, 4 additional articles were identified from the lists of references, resulting in a total of 32 articles eligible for the final systematic review. The detailed PRISMA flowchart of inclusion/exclusion of potential publications is presented in **Figure 1**.

### Study Characteristics

**Table 1** describes the characteristics of the included studies including 14 for the association between TMI and body fat in childhood and adolescence (10, 14, 18–26, 41–43), 20 for TMI and CVRFs in childhood and adolescence (13–18, 21, 26–34, 42–45), and 4 for the association in adulthood (35–37, 46). Twenty-five of the included studies used a cross-sectional design (10, 13, 15–28, 30–34, 41–43, 45), 2 a retrospective design (14, 44), 1 a case-control design (29), and 4 a cohort design (35–37, 46).

**Table 1:** Study characteristics of the included studies.

Outcome	First author,year	Study name	Country of origin and design	Study design	Age, years	Sample size	Sex: (male, %)	Exposures	Outcome definition
**Body fat**									
	Peterson, 2017 (10)	The 1990–2006 US National Health and Nutrition Examination Survey	USA	Cross-sectional	8–17	2,285	55.1	TMI *vs*. BMI	Continuous: BF% by DXA Categorical: Overweight was based on the 85th–95th percentiles of TMI, BMI, and BF%
	Jiang, 2018 (18)	A multicentre cross-sectional study in east and southwest China	China	Cross-sectional	7–18	1,860	49.7	TMI *vs*. WHtR, BMI SDS, WC SDS	Continuous: BF% by DXA
	Sims, 2018 (19)	The Canadian Study of Determinants of Endometabolic Health in China	Canada	Cross-sectional	5–17	181: 44 SCBT and 137 non-cancer control children	53.6	TMI *vs*. BMI	Continuous BF% by bioelectrical impedance, WHtR, WHpR
	Akcan, 2019 (14)	Study from the Pediatric Endocrinology Outpatient Clinics of the Near East University	Cyprus	Retrospective	6.3–17.6	143	42	TMI *vs*. BMI	Overweight: BMI-SDS +1.0 to +2.0; TMI 16.8 kg/m^3^ for girls and 16.0 kg/m^3^ for boys obesity: BMI-SDS ≥+2.0; TMI 19.7 kg/m^3^ for girls and 18.8 kg/m^3^ for boys
	Moselakgomo,2019 (20)	Study from the Limpopo and Mpumalanga province of South Africa	South African	Cross-sectional	9–13	1,361	49.8	TMI *vs*. BMI	Overweight and obesity were based on age- and sex-specific TMI and BMI percentages of the study population
	Ashley-Martin, 2019 (21)	Canadian Health Measures Survey	Canada	Cross-sectional	6–19	5,814	50.7	TMI *vs*. BMI	Overweight and obesity: based on BMI z-score of the International Obesity Task Force and age- and sex-specific 85th and 95th TMI percentiles of the National Health and Nutrition Examination Survey.
	Zaniqueli, 2019 (22)	Study from the municipality of Serra and Vitória, State of Espírito Santo, Brazil	Brazil	Cross-sectional	6–18	1,149	53.2	TMI *vs*. BMI	BF% was by bioelectrical impedance. Obesity: respectively based on the 95th percentile of TMI, BMI, and BF%
	De Lorenzo, 2019 (23)	Study from the University of Rome Tor Vergata, Human Nutrition Unit, Italy	Italy	Cross-sectional	8–17	485	42.7	TMI *vs*. BMI	BF% by DXA High adiposity: ≥75^th^ percentile of BF%
	Nascimento, 2019 (24)	Study from Taubaté, São Paulo, Brazil	Brazil	Cross-sectional	2–5	919	50.1	TMI *vs*. BMI	WHtR was used to define central fat accumulation: the upper tertile of the study population
	Woolcott, 2019 (25)	The National Health and Nutrition Examination Survey from 1999 to 2006	USA	Cross-sectional	8–19	10,390	56.8	TMI *vs*. RFMp (RFM), BMI, WHtR	BF% by DXA Overweight and obesity diagnoses using BMI, TMI, RFMp, RFM, and WHtR were defined based on 85th and 95th percentiles, respectively (BMI specific for sex and age, the others specific for sex).
	Park, 2020 (26)	Korea National Health and Nutrition Examination Survey, 2007–2016	Korea	Cross-sectional	10–20	9,749	51.5	TMI *vs*. BMI	Overweight: BMI or TMI was ≥85th percentile and <95th percentile Obesity: BMI or TMI was ≥95th percentile
	Ye, 2020 (41)	Data from the Qibao Community in Minhang District of Shanghai	China	Cross-sectional	6–17	14,042	54.3	TMI *vs*. BMI, WHtR, WHR, WC, body adiposity index	BF% measured using bioelectrical impedance analysis (boys aged 6–18 years: ≥20%; girls aged 6–14 years: ≥25%; girls aged 15–18 years: ≥30%)
	Alfaraidi 2021 (42)	The Improving Renal Complications in Adolescents with Type 2 Diabetes Through Research cohort Study	Canada	Cross-sectional	10.2–17.9	116	31.0	TMI *vs*. BMI z-score	FM% and WHtR
	Malavazos 2021 (43)	The Italian “Educazione A limentare Teenagers” project survey	Italy	Cross-sectional	12–13	3479	54.3	TMI *vs*. BMI or BMI z-score	Central obesity was defined as WHtR ≥0.5
**Obesity-related cardiovascular risk factors**									
	Ramírez-Vélez, 2018 (27)	The Fuprecol Study in Bogotá, Colombia	Columbia	Cross-sectional	9–25	4673	42.9	TMI *vs*. FMI	MetS was defined as 3 or more of following criteria (1): abdominal obesity: WC ≥90 cm for men and 80 cm for women; (2) hypertriglyceridemia: ≥150 g/dl; (3) low HDL-C: <40 mg/dl for men and <50 mg/dl for women; (4) high BP: ≥130/85 mmHg; (5) high fasting glucose: ≥100 mg/dl.
	Gomes, 2018 (15)	Study from the North and Central regions of mainland Portugal	Portugal	Cross-sectional	10–17	1,324	47.1	TMI *vs*. BMI, BMI z-score, WC, WC/H, and WC/Hadj.	A standardized metabolic risk score was computed by summing of standardized values for fasting glucose, triglycerides, high-density lipoprotein cholesterol, and mean arterial BP.
	Jiang, 2018 (18)	A multicentre cross-sectional study in east and southwest China	China	Cross-sectional	7–18	1,860	49.7	TMI *vs*. WHtR, BMI SDS, WC SDS, and BF%	CMR1 and CMR2 were defined as 3 or more and 2 or more following abnormalities: (1) Hypertension: based on age- and sex-specific reference of Chinese children and adolescents, (2) Dyslipidemia: TG ≥ 1.76 mmol/l or TC ≥ 5.2 mmol/l or LDL-C ≥ 3.38 mmol/l, or HDL-C ≤ 1.04 mmol/l, (3) Elevated fasting blood glucose ≥ 5.6 mmol/l, (4) Central obesity: recommended by the China children’s obesity working group.
	Ashley-Martin, 2019 (21)	The Canadian Health Measures Survey	Canada	Cross-sectional	6–19	5,814	50.7	TMI *vs*. BMI	High TC: ≥ 200 mg/dl, low HDL-C: <40 mg/dl, TG ≥ 100 mg/dl for 0–9 years and ≥130 mg/dl for 10–19 years, C-reactive protein: >3.0 mg/l, HOMA-IR: ≥90th percentile, and high BP: SBP and/or DBP ≥90th percentile.
	Shim, 2019 (28)	Korea National Health and Nutrition Examination Survey, 2007–2016.	Korea	Cross-sectional	10–20	8,464	51.6	TMI	MetS was defined as 1 or more of the following criteria: (1) elevated WC: ≥90th percentile, (2) elevated BP: ≥90th percentile, (3) elevated glucose: ≥110 mg/dl, (4) elevated TGs:≥110 mg/dl, and (5) reduced HDL-C: <40 mg/dl.
	Akcan, 2019 (14)	Study from the Pediatric Endocrinology Outpatient Clinics of the Near East University	Cyprus	Retrospective	6.3–17.6	143	42.0	TMI *vs*. BMI	IR: Prepubertal girls: 2.22; prepubertal boys: 2.67; pubertal girls: 3.82; and pubertal boys: 5.22 High liver enzymes: The threshold for serum glutamic oxaloacetic transaminase: 5–34 U/l, serum glutamic pyruvic transaminase: 0–55 U/l Dyslipidemia: TC ≥ 200 mg/dl; TG ≥ 150 mg/dl; HDL < 40 mg/dl; and LDL ≥ 100 mg/dl
	Arsang-Jang, 2019 (30)	The Adolescence Surveillance and Prevention of Adult Non-communicable disease survey	Iran	Cross-sectional study	7–18	24,409	50.1	TMI *vs*. BMI, TBSI, WC, WH.5R, WHtR	MetS: abdominal obesity plus at least 2 of the following risk factors: (1) high TG ≥ 150 mg/dl; low HDL-C: males, <40 mg/dl and females, <50 mg/dl; high BP, SBP/DBP ≥ 130/85 mm Hg; high FPG: ≥100 mg/dl or previously diagnosed as T2DM
	Radetti, 2019 (16)	Study from the obesity inpatient clinic of the Istituto Auxologico Italiano, Piancavallo, Verbania, Italy	Italy	Cross-sectional	10–17	1,332	41.6	TMI *vs*. BMI, BMI SDS, FFMI, FMI, WHtR, BMFI	Mets: abdominal obesity plus at least 2 of the following risk factors: (1) high TG ≥ 150 mg/dl; low HDL-C: males, <40 mg/dl and females, <50 mg/dl; high BP, SBP/DBP ≥ 130/85 mm Hg; high FPG: ≥100 mg/dl or previously diagnosed as T2DM
	Umano, 2019 (31)	Obesity outpatient clinic in Italy	Italy	Cross-sectional	4–18	1,387	51.4	TMI *vs*. BMI z-score, WC z-score and WHtR	BP, glucose, insulin, and lipid profile
	Wang, 2020 (13)	A Chinese National School-based Health Survey and United States National Health and Nutrition Examination Survey	China and the USA	Cross-sectional	7–18 for Chinese; 12–18 for American	57,201 Chinese children and 10,441 American children	51.6 for Chinese; 50.9 for American	TMI *vs*. BMI, BMI z-score, weight/height^2.5^	Impaired FPG:≥5.6 mmol/l; dyslipidemia: TC ≥ 170 mg/dl; high LDL-C: ≥110 mg/dl; low HDL-C: <120 mg/dl; TG ≥ 75 mg/dl for children under 9 years and ≥90 mg/dl for children more than 10 years; HBP: BP ≥ 90th percentile
	Park, 2020 (26)	Korea National Health and Nutrition Examination Survey, 2007–2016.	Korea	Cross-sectional	10–20	9,749	51.5	TMI *vs*. BMI	DBP, SBP, HDL-C, LDL-C, TC, TG, WC
	Akcan, 2020 (29)	Study from the Pediatric Endocrinology Outpatient Clinics of the Near East University	Cyprus	Case–control study	5.3–17.4	80	42.5	TMI *vs*. BMI	IR: prepubertal girls: 2.22; prepubertal boys: 2.67; pubertal girls: 3.82; and pubertal boys: 5.22; Low HDL-C: <40 mg/dl; High TG: >150 mg/dl
	Matsuo, 2020 (32)	Study on the effectiveness of multidisciplinary obesity treatment program in Brazil	Brazil	Cross-sectional	12–18	217	38.7	TMI *vs*. BMI, WC, WHtR	HOMA-IR: cutoff point of ≤3.16
	Khoshhali, 2020 (33)	The fifth survey of “Childhood and Adolescence Surveillance and Prevention of Adult Non-communicable Disease”	Iran	Cross-sectional	7–18	3731	52.6	TMI *vs*. BMI	MetS was defined as 3 or more of following criteria: (1) abdominal obesity: WHtR ≥0.5, (2) elevated FBG: ≥100 mg/dl, (4) high TG: ≥100 mg/dl, (5) low HDL-C: <40 mg/dl, (6) elevated BP: ≥ age-, sex-, and height-specific 90th percentile
	Neves, 2020 (34)	Study from the Vitória, Espírito Santo, Brazil	Brazil	Cross-sectional	8–14	296	45.6	TMI *vs*. BMI z-score	HOMA-IR: based on *β*-cell function (%) = 20*insulin/(glucose-3.5); resistance = insulin/(22.5e^-lnglucose^)
	Leone, 2020 (17)	International Center for the Assessment of Nutritional Status	Italy	Cross-sectional	7–20	403	44.4	TMI *vs*. BMI z-score, WHtR, body shape index z-score, and conicity index	MetS: 7–10 years (three or more of the following criteria: WC ≥ 90th percentile; systolic or diastolic BP ≥ 90th percentile; TG ≥ 90th percentile or HDL ≤ 10th percentile; HOMA-IR ≥ 90th percentile or FPG ≥ 90th percentile; 10–20 years: IDF criteria
	Umano, 2020 (44)	A study from an obesity outpatient clinic of the Department of Pediatrics of the University of Campania Luigi Vanvitelli of Naples	Italy	Retrospective study	10.5 ± 2.89	1,900	50.2	TMI *vs*. BMI z-score and WHR	Non-alcoholic fatty liver disease was assessed based on high-level and abnormally intense echoes from the liver kidney and hepatic parenchyma in echo amplitude
	Alfaraidi, 2021 (42)	Improving Renal Complications in Adolescents with Type 2 diabetes Through Research cohort study	Canada	Cross-sectional	10.2–17.9	116	31.0	TMI *vs*. BMI z-score	HDL
	Calcaterra 2021 (45)	Outpatient clinics in Milan	Italy	Cross-sectional	6–18	585	47.7	TMI *vs*. BMI or BMI z-score	HOMA-IR; HOMA-β; quantitative insulin sensitivity check index; triglyceride and glucose index
	Malavazos 2021 (43)	The Italian “Educazione A limentare Teenagers” project survey	Italy	Cross-sectional	12–13	3,479	54.3	TMI *vs*. BMI or BMI z-score	BP ≥ age-, sex-, and height-specific 90th percentile of the NHBPEP Working Group
**Adult health conditions**									
	Wu, 2018 (1) (36)	The Childhood Determinants of Adult Health Study	Australia	Cohort	7–15 at baseline	2,345	49.1	TMI *vs*. WC, WC adjusted for height, weight adjusted for height, HC, waist–hip ratio, WHtR, BMI, conicity index, AVI, body adiposity index, and a body shape index.	HOMA2-β: beta-cell function and fasting insulin ≥75th percentile; HOMA-IR: HOMA index ≥75th percentile; High fasting insulin:≥ 5.6 mmol/l
	Wu, 2018(2) (35)	The Cardiovascular Risk in Young Finns Study	Finland	Cohort	3–18 at baseline	2,626	–	TMI and its combination with BMI or SST *vs*. BMI	T2D: FPG ≥ 126 mg/dl or hemoglobin A1c ≥6.5%, or used glucose-lowing medication; obesity: BMI ≥ 30 kg/m^2^; Hypertension: SBP and/or DBP ≥ 140/90 mmHg, abnormal LDL-C: ≥160 mg/dl, HDL-C:<40 mg/dl, and high carotid intima-media thickness: ≥90th percentiles
	Wu, 2020 (37)	The ongoing Special Turku Coronary Risk Factor Intervention Project	Finland	Cohort	2–20	432	48.1	TMI *vs*. BMI	Aortic intima-media thickness, IFG, elevated insulin levels, HOMA-IR, serum lipids, and hypertension
	Wu, 2021 (46)	Taipei City Hospital Radiation Building Database	Taiwan (China)	Cohort	13–18	1,387	49.7	TMI *vs*. BMI-z score	Diabetes: FPG ≥ 126 mg/dl or diagnosed by physicians or current use of diabetes medicine

AUC, area under the curve; AVI, abdominal volume index; BMI, body mass index; BP, blood pressure; BMFI, body mass fat index; CMR, cardiometabolic risk; FPG, raised fasting plasma glucose; FMI, fat mass index; FFMI, fat-free mass index; FMI, fat mass index; HC, hip circumference; HOMA-IR, homeostasis model assessment-insulin resistance; HOMA2-β, homeostasis model assessment of beta-cell function; HOMA2-IR, homeostasis model assessment of insulin resistance; HDL-C, high-density lipoprotein cholesterol; IFG, impaired fasting glucose; IR insulin resistance; LDL-C, low-density lipoprotein cholesterol; MetS, metabolic syndrome; RFMp: relative fat mass pediatric; SCBT, survivors of childhood brain tumors; SD, standard deviation; TMI, tri-ponderal mass index; TC, total cholesterol; T2D, type 2 diabetes; TBSI, tri-ponderal body shape index; TG, triglycerides; WC, waist circumference; WC/H, WC/height ratio; WC/Hadj, WC/H adjusted ratio; WH.5R, WC to height 5; WHtR, waist-to-height ratio; WHR, waist to hip ratio; FM%, percent of fat mass; SGOT, serum glutamic oxaloacetic transaminase; SGPT, serum glutamic-pyruvic transaminase; SST, subscapular skinfold thickness.

### Results

As shown in **Table 2**, All of the 32 included studies were of moderate to high quality except for one article rated as having a low quality (quality score = 3) (14).

**Table 2:** Results of the included studies.

Outcome	First author,year	Results	Adjusted covariates	Study quality
**Body fat**				
	Peterson, 2017 (10)	(1) For children and adolescents aged 8 to 17 years, TMI was better to estimate BF% than BMI, especially in boys (boys: R^2^ = 0.64 *vs*. 0.38; girls: R^2^ = 0.72 *vs*. 0.66). (2) The misclassification of overweight was less than BMI z-score (8.4% *vs*. 19.4%, p < 0.001) but equal to updated BMI percentiles based on the same data set (8.4% *vs*. 8.0%, p = 0.62). However, TMI was preferred due to its simplicity with no complicated percentiles. (3) The results were similar when stratified by sex.	None	6
	Jiang, 2018 (18)	(1) WHtR was most strongly correlated with BF% (*rho* coefficient =0.73, *p* < 0.001), followed by WC SDS, TMI, and BMI SDS (*rho* =0.71 *vs*. 0.68 *vs*. 0.68, *p* < 0.001). (2) TMI and WHtR were more applicable for public health use than BMI, WC, and BF% due to their simplicity in calculating and identifying obesity. The AUCs of these indicators remained similar when stratified by sex.	None	6
	Sims, 2018 (19)	After adjusting for potential variables, the correlation between TMI and BF% was equal to BMI z-score (*r*= 0.85 *vs*. 0.85), whereas the correlation between TMI and WHpR (*r* = 0.46 *vs*. 0.41) or WHtR (*r*= 0.86 *vs*. 0.78) was stronger than BMI z-score.	Age, sex, treatment, and puberty	6
	Akcan, 2019 (14)	TMI revealed less overweight and obesity than BMI. About 22 overweight children and 8 obese children identified by BMI-SDS were regarded as normal-weight children identified by TMI. 44 obese children (based on BMI) were overweight according to TMI.	None	3^*^
	Moselakgomo, 2019 (20)	TMI revealed more overweight and obesity than BMI (overweight: 5.66% *vs*. 1.84%; obesity: 1.98% *vs*. 0.66%). The classification of overweight and obesity by TMI and BMI were as follows: overweight: boys: 7.3% *vs*. 2.6%; 2.2% *vs*. 0.7%; girls: 4.0% *vs*. 1.0%; obesity: boys: 2.2% *vs*. 0.7%; girls: 1.8% *vs*. 0.6%.	None	5
	Ashley-Martin, 2019 (21)	The prevalence of overweight defined by TMI was lower than that defined by BMI (15% *vs*. 18%), but the prevalence of obesity defined by TMI was higher than that defined by BMI (9.7% *vs*. 8.9%)	None	4
	Zaniqueli, 2019 (22)	Although TMI (R^2 =^ 0.73 for boys and R^2 =^ 0.75 for girls) and BMI (R^2^ = 0.74 for boys and R^2^ = 0.75 for girls) performed similar in the portion of the variability for BF%, TMI was recommended to replace the BMI z-score in children and adolescents due to a lower false-positive rate of obesity (boys: 21.8% *vs*. 3.9%; girls: 28.5% *vs*. 17.5%).	None	6
	De Lorenzo, 2019 (23)	TMI was a better predictor for BF% in both sexes than BMI (boys: R^2^ = 0.67 *vs*. 0.44; girls: R^2^ = 0.79 *vs*. 0.74). TMI presents a higher AUC value than BMI for predicting high adiposity in children and adolescents (0.96 *vs*. 0.93).	None	5
	Nascimento, 2019 (24)	The AUC of TMI was higher than BMI for screening central fat accumulation (0.92 *vs*. 0.87), regardless of sex.	None	6
	Woolcott, 2019 (25)	(1) RFMp and WHtR showed similar linear association with BF%, followed by TMI and BMI in children and adolescents 8 to 14 years (R^2^ = 0.77, 0.76, 0.69, 0.55 for boys, R^2^ = 0.74, 0.74, 0.71, 0.65 for girls). (2) Similar results in boys aged 15 to 19 years. WHtR (R^2^ = 0.80 for boys and 0.70 for girls) showed higher predicting ability than RFM (0.79 for boys and 0.72 for girls) among boys, followed by BMI and TMI in children and adolescents aged 15-19 years (R^2^ = 0.70 and 0.69 for boys and 0.73 and 0.72 for girls. However, the predicting ability was similar among girls. (3) RFMp for children and adolescents 8 to 14 years of age and RFM for adolescents 15 to 19 years of age were useful to estimate whole-body fat percentage and diagnose body fat-defined overweight or obesity.	None	6
	Park, 2020 (26)	The prevalence of overweight defined by TMI was slightly higher than that defined by BMI (10.6% *vs*. 10.2%), but the prevalence of obesity defined by TMI was lower than that defined by BMI (5.3% *vs*. 10.6%), similar in both sex.	None	5
	Ye, 2020 (41)	The correlation between BMI and BF% (r = 0.919) was higher than TMI (r = 0.896), WC (r = 0.842), WHtR (r = 0.830), and WHR (r = 0.522). For children aged 6–11 years, the AUC values of BMI (0.980 for boys and 0.981 for girls) was significantly higher than TMI (0.957 and 0.948), WC (0.940 and 0.945), and WHtR (0.939 and 0.921) whereas for adolescents aged 12–17 years, TMI (0.976 for males and 0.945 for females) performed better than BMI (0.967 and 0.943), WHtR (0.960 and 0.878), and WC (0.945 and 0.864) to identify obesity	Age and sex	8
	Alfaraidi, 2021 (42)	TMI was associated with FM% (r = 0.74, *p* < 0.0001) and WHtR (r = 0.85, *p* < 0.0001), among adolescents with type 2 diabetes, whereas BMI was not.	Age and sex	8
	Malavazos, 2021 (43)	TMI was better than BMI and BMI z-score to discriminate central fat among adolescents. (AUC in boys: TMI 0.96, BMI, 0.95, *p* < 0.001, BMI z-score 0.95, *p* = 0.002; AUC in girls: TMI 0.97, BMI 0.96, *p* < 0.0001, BMI z-score 0.96, *p* < 0.0001)) The prevalence of central obesity based on TMI (96.6% in boys and 97.3% in girls) was higher than BMI (50.7% in boys and 34.6% in girls) and BMI z-score (52.4% in boys and 38.7% in girls) among adolescents with overweight	None	7
**Obesity-related cardiovascular risk factors**				
	Ramírez-Vélez, 2018 (27)	The power of TMI to detect MetS was comparable to FMI 9–12 years AUCs for girls: TMI: 0.674; FMI: 0.698. AUCs for boys: TMI: 0.755; FMI: 0.752. 13–17 years AUCs for girls: TMI: 0.684; FMI: 0.699. AUCs for boys: TMI 0.729; FMI: 0.745	None	5
	Gomes, 2018 (15)	BMI z-score (AUC 0.678), BMI (0.683), and WC (0.676) were a stronger predictor for metabolic risk score than TMI (0.655)	None	4
	Jiang, 2018 (18)	TMI showed similar good performance in identifying CMR (AUC of CMR1 and CMR2: 0.88, 95% CI 0.84–0.92; 0.82, 0.79–0.85) to WHtR (0.88, 0.83–0.92; 0.82, 0.79–0.86), BMI SDS (0.89, 0.85–0.93; 0.84, 0.81–0.87), and WC SDS (0.89, 0.85–0.93; 0.84, 0.81–0.87), but higher performance than BF% (0.83, 0.78–0.88; 0.77, 0.74–0.80).	None	6
	Ashley-Martin, 2019 (21)	Similar to BMI, TMI was a good predictor for HOMA-IR or having more than 3 abnormal tests (AUC 0.83 and 0.81), but poor for CRP (0.73 and 0.74), high TG (0.67 and 0.68), low-HDL-C (0.67 and 0.66), high TC (0.60 and 0.62), and high BP (SBP: 0.66 and 0.66; DBP: 0.55 and 0.56).	None	4
	Shim, 2019 (28)	Compared with normal weight, overweight defined by TMI was associated with MetS (OR 25.57) and its components, including low HDL-C (2.31), elevated TG (2.55), elevated BP (1.33), and elevated WC (29.18). The association was stronger for obesity defined by TMI, suggesting TMI might be used as a screening tool for overweight and obesity in a clinical setting.	Age, sex, alcohol consumption, smoking, household income, physical activity, rural residence, hypertension, diabetes mellitus, and dyslipidemia	7
	Akcan, 2019 (14)	IR: Compared to BMI, TMI was more likely to overlook IR. Of 22 overweight children defined by BMI with normal TMI, 22.7% had IR. 2 of 8 obese children (25%) defined by BMI with normal TMI had IR. Among 44 obese children based on BMI but overweight based on TMI and 40.9% were detected as IR. High Liver enzymes: Compared to BMI, TMI was better to predict visceral adiposity High liver enzymes were not found in any of the children with normal TMI. Dyslipidemia: Among overweight children based on BMI but normal with TMI, 9.1% had high TC, 4.5% high TG and low HDL-C, and 50% high LDL	None	3^*^
	Arsang-Jang, 2019 (30)	Among adolescents, compared with BMI, TMI, WC, WHtR, and WH.5R, the TBSI (WC z-score/(TMI^2/3^*Height^1/2^) was considered the best predictor of MetS. The TBSI was significantly more accurate than the BMI and TMI (Youden index: 0.85 *vs*. 0.73 *vs*. 0.70) for classifying individuals with MetS and in healthy groups.	None	5
	Radetti, 2019 (16)	BMFI (BMI*FM% *WC; AUC female, 0.69; male 0.59) performed marginally better than BMI (0.68 and 0.58), TMI (0.66 and 0.55), FMI (0.67 and 0.58), FFMI (0.61 and 0.55), WHtR (0.68 and 0.56), and BMI SDS (0.68 and 0.58) in predicting MetS	None	6
	Umano, 2019 (31)	WHtR performed best in predicting lipid metabolism markers and glucose, followed by the TMI, WC z-score, and BMI z-score among children and adolescents with obesity.	Age, gender, and pubertal stage	7
	Wang, 2020 (13)	(1) TMI was significantly associated with metabolic variables, the ranges of ORs were 1.09 (95% CI 1.04, 1.14) for impaired FPG, 1.13 for dyslipidemia (95% CI 1.11, 1.15), and 1.23 (95% CI 1.22, 1.25) for high BP. Similar results were found among Americans. (2) TMI showed similar values to BMI percentiles but were more precise than BMI z-score to predict cardiovascular risks. However, for specific cardiovascular risks, TMI was similar to BMI to identify IR, better than BMI to identify high BP, and poor as BMI to identify dyslipidemia. (3) The ranges of misclassification rates were 19.1% to 34.7% for TMI and 26.3% to 36.8% for BMI z-score in Chinese, similar for American subjects.	Age and sex	8
	Park, 2020 (26)	(1)Among those with normal BMI, boys with overweight TMI had higher TC (174.4 mg/dl *vs*. 153.6 mg/dl, *p* = 0.002) and TG (101.9 mg/dl *vs*. 77.4 mg/dl, *p* = 0.028), compared with boys with normal TMI; girls with overweight TMI had lower HDL-C (50.1 mg/dl *vs*. 53.5 mg/dl, *p* = 0.045) and higher TG (102.8 mg/dl *vs*. 81.4 mg/dl, *p* = 0.029), compared with girls with normal TMI. (2) Among those with overweight BMI, boys with overweight TMI had higher TC (169.8 mg/dl *vs*. 157.5 mg/dl, *p* < 0.001) and LDL-C (101.7 mg/dl *vs*. 90.8 mg/dl, *p* < 0.001), girls had lower HDL-C (49.5 mg/dl *vs*. 51.9 mg/dl, *p* = 0.013) and TG (96.5 mg/dl *vs*. 82.6 mg/dl, *p* = 0.004), compared with those with normal TMI. (3) The obesity-related comorbidities (except for DBP) of the overweight group (based on TMI) were worse under the same BMI category (normal or overweight).	None	6
	Akcan, 2020 (29)	TMI was associated with a similar amount of metabolic markers to BMI. BMI as a continuous variable seemed to be more strongly associated with TC (R^2^: 0.32 *vs*. 0.27), HDL (-0.52 *vs*. -0.46), and TG (0.32 *vs*. 0.27) and TMI was more strongly associated with low-density lipoprotein-cholesterol (LDL-C) (0.38 *vs*. 0.33). Leptin, IL-6, and fetuin-A were more closely correlated with BMI than TMI	None	4^*^
	Matsuo, 2020 (32)	(1) In overweight adolescents, WC presented the most predictive capacity to explain IR and BMI had a slightly better predictive capacity than TMI, regardless of sex. (2) In boys, TMI and BMI showed similar values of sensibility (88.4% *vs*. 88.2%) and specificity (42.4% *vs*. 45.5%). Nevertheless, BMI had a better sensibility (57.1% *vs*. 49.0%) while TMI had a better specificity (88.1% *vs*. 81.0%) for girls. WC demonstrated a strong sensibility (boys: 82.4%; girls: 79.6%) for both sexes.	None	6
	Khoshhali, 2020 (33)	Among boys, the AUC in identifying MetS of TMI was similar to BMI for both 7–10 years (0.72 *vs*. 0.69), 15–18 years (0.70 *vs*. 0.67), 11–14 years (0.74 *vs*. 0.74), and 7–18 years (0.72 *vs*. 0.69), as well as among girls at age 7–18 years (AUC = 0.68 *vs*. 0.67)	None	5
	Neves, 2020 (34)	TMI showed a similar performance in identifying HOMA-IR to BMI z-score for both sex (boys: TMI = 0.843, BMI z-scores = 0.831; girls: TMI = 0.763, BMI z-scores = 0.756).	None	4
	Leone, 2020 (17)	MetS Children aged <10 years: only BMI z-score was associated with MetS (*β* = 2.21, *p* < 0.05) Children aged ≥10 years: BMI z-score (*β* = 2.67, *p* < 0.001), TMI (*β* = 0.19, *p* < 0.01), conicity index (*β* = 9.02, *p* < 0.001) and WHR (*β* = 12.32, *p* < 0.001) were associated with MetS. Similar results were found among males, whereas only conicity index (*β* = 7.37, *p* < 0.05) and WHR (*β* = 7.94, *p* < 0.05) were associated with MetS among females. High BP: BMI z-score was the best predictor of high BP in both children and adolescents, whereas TMI performed better among males. High TG: conicity index was the best predictor for high TG in females and WHR was best in males. Low HDL-C: BMI z-score was the best indicator for low HDL-C.	Age and sex	8
	Umano, 2020 (44)	The AUC of WHR (0.62) was higher than TMI (0.58) and BMI (0.58).	None	7
	Alfaraidi, 2021 (42)	TMI was associated with HDL (r = -0.26, *p* < 0.005) among adolescents with type 2 diabetes, whereas BMI was not.	Age and sex	8
	Calcaterra, 2021 (45)	Among children and adolescents with obesity, TMI was associated with IR indicators only in females while BMI correlated with all IR indicators except for triglyceride and glucose index in females and BMI z score correlated with all IR indicators except for HOMA-β in males.	None	7
	Malavazos 2021 (43)	TMI was better than BMI and BMI z-score to discriminate hypertension. (AUC in boys: TMI 0.73, BMI, 0.70, *p* = 0.002, BMI z-score, 0.70, *p* = 0.020; AUC in girls: TMI 0.76, BMI 0.73, *p* = 0.002, BMI z-score, 0.74, *p* = 0.020)	None	7
**Adult health conditions**				
	Wu, 2018a (36)	TMI of children was significantly correlated with adult HOMA2-IR (RR 1.15, 95% CI 1.02, 1.29), high HOMA2-β (RR 1.25, 95% CI 1.11, 1.40), and high fasting insulin (RR 1.17, 95% CI 1.04, 1.31). However, the predictive ability was low with AUCs of 0.53, 0.56, and 0.54, respectively, which was lower than other indicators such as abdominal volume index, BMI, and WC.	None	7^*^
	Wu, 2018b (35)	(1) Youth TMI, BMI, and subscapular skinfold thickness were significantly associated with adult T2D, obesity, high carotid intima-media thickness, and high LDL-C level. (2) Youth TMI was not associated with adult hypertension and low HDL-C (3) Youth BMI was superior or comparable to TMI and SST in predicting adult T2D (AUC 0.688 *vs*. 0.682 *vs*. 0.683), obesity (0.726 *vs*. 0.673 *vs*. 0.683), hypertension (0.660 *vs*. 0.656 *vs*. 0.660), high carotid intima-media thickness (0.568 *vs*. 0.554 *vs*. 0.557), and high LDL-C level (0.609 *vs*. 0.608 *vs*. 0.614).	None	7^*^
	Wu, 2020 (37)	(1) BMI had stronger associations with insulin (at age 16 years), SBP (age 5–20 years), and TG (age 18 years) than TMI. (2) Between the ages of 14 and 16, BMI outperformed TMI for elevated insulin levels (difference in AUC = 0.018 and 0.025) and IR (difference in AUC = 0.018–0.024). At age 16–20 years, BMI outperformed TMI for hypertension (difference in AUC = 0.017–0.022). For other outcomes of impaired FPG, high aortic intima-media thickness, high LDL-C, low HDL-C, and high TG, the predictive utilities were similar.	None	7^*^
	Wu, 2021 (46)	Persistent increase of TMI during 13–18 years was associated with increased risk of diabetes in adulthood (hazard ratio: 2.85, 95% confidence interval: 1.01–8.09). No association was found for BMI z score (2.79, 0.35–22.00)	Age, sex, baseline weight status, height, family history of diabetes, smoking, systolic and diastolic BP, TG, and fasting glucose cholesterol	8

*The study quality was assessed by Newcastle-Ottawa Scale and others were assessed by Agency for Healthcare Research and Quality.

#### TMI for Screening Body Fat in Children and Adolescents

A total of 14 articles evaluated the ability of TMI to identify body fat mass in children and adolescents compared with BMI (**Table 2**) (10, 14, 18–26, 41–43).

It has been shown that percent of body fat (BF%) as a gold standard was better predicted by TMI than by BMI (10, 25), although one study reported that both relative fat mass pediatric (RFMp) based on height and waist circumference [WC], and waist-to-height ratio (WHtR) performed better than both TMI and BMI (25). When WHtR was used to define central obesity as the gold standard, three articles showed that TMI was better than BMI correlated with central fat accumulation in both preschool-aged children aged 2–5 years (24) and children and adolescents aged 5–17 years (19, 43).

When BMI and TMI were used as continuous variables, TMI correlated similarly or better than BMI with BF% in children and adolescents (18, 19, 22, 23, 41, 42). Although TMI and BMI among children and adolescents aged 5–18 years explained a similar proportion of the variability for BF%, TMI was recommended to replace BMI z-score in children and adolescents due to its lower false-positive rate of obesity than the BMI z-score (boys: 2.9% *vs*. 21.8%; girls: 17.5% *vs*. 28.5%) (18, 19, 22). TMI presented a higher area under the curve (AUC) value than BMI for predicting high BF% (0.96 *vs*. 0.93, *p* < 0.001) measured by dual-energy X-ray absorptiometry (DEXA) among children and adolescents aged 8–17 years (23) or more strongly correlated with BF% compared to BMI in adolescents (41, 42).

The remaining four studies could not conclude about a possible advantage of either TMI or BMI to identify overweight or obesity status because of the lack of a gold standard (to objectively assess adiposity) and inconsistent cutoffs (14, 20, 21, 26). Akcan et al. found that TMI identified a lower prevalence of overweight and obesity among children aged 6–17 years compared to BMI-SDS (14), which was contrary to the finding among children aged 9–13 years, independent of sex (20). When considering overweight and obesity separately, Ashley-Martin et al. found that BMI defined more overweight than TMI, whereas TMI defined more obesity than BMI among children and adolescents aged 6–19 years (21), inversely to the findings among children and adolescents aged 10–20 years reported by Park et al. (26).

Overall, studies using a gold standard for comparison and using BMI and TMI as continuous variables suggested that TMI performed equally or better than the widely used BMI to predict BF% and central fat among children and adolescents. TMI was preferred in adolescence due to its stability.

#### TMI and Obesity-Related Cardiovascular Risk Factors in Children and Adolescents

Twenty articles on the association between TMI and MetS and its components were included in this systematic review (**Table 2**) (13–18, 21, 26–34, 42–45).

##### MetS

Ten articles have evaluated the association of TMI and other anthropometric indicators with MetS, metabolic risk score, or cardio-metabolic risk (13, 15–18, 27–30, 33). Three of the 10 articles showed that TMI was not better than other indicators such as BMI (or BMI z-score or BMI-SDS) among children and adolescents aged 10–17 years to predict MetS and a metabolic risk score (15–17). However, the other seven articles suggested that TMI could be a useful screening tool or similar to BMI in predicting MetS or cardio-metabolic risks in children and adolescents aged 5.3 to 25 years (13, 18, 27–30, 33).

TMI was found to be associated with obesity-related CVRFs, including MetS and its components [elevated blood pressure (BP), elevated WC, low high-density lipoprotein cholesterol (HDL-C), and elevated triglycerides (TG)] in late adolescence (28). It was reported that TMI performed similarly to FMI (27) or BMI, or was an auxiliary indicator in addition to BMI, to identify MetS, a metabolic risk score, or CVRFs among children and adolescents aged 5–18 years (13, 18, 29, 33). However, the tri-ponderal body shape index [WC z-score/(TMI^2/3^*height^1/2^)] including TMI and WC z-score components performed more accurately in predicting MetS than BMI and TMI (Youden index: 0.85 *vs*. 0.73 *vs*. 0.70) among children and adolescents aged 7–18 years, suggesting that the combination of TMI and a WC z-score could be considered as a useful predictor for MetS in children and adolescents (30).

Overall, TMI performed similarly as compared to BMI and other indicators in predicting MetS in most of the included studies, and TMI was also suggested to be a useful tool when used in combination with other adiposity indicators (e.g., BMI and WC) for identifying MetS.

##### Insulin Resistance

Eight articles compared TMI and BMI for identifying insulin resistance (IR) or impaired glucose in children and adolescents (**Table 2**) (13, 14, 21, 29, 31, 32, 34, 45). Among these eight articles, seven reported that BMI (used as a continuous variable) performed similarly or marginally better than TMI for identifying IR (13, 21, 29, 31, 32, 34, 45). In addition, compared to BMI, TMI was more likely to underestimate IR (14). The inconsistent cutoffs of TMI and BMI for identifying overweight might lead to different identification of IR. When restricted to children and adolescents aged 4–18 years with overweight or obesity, WHtR or WC, used as continuous variables, seemed to perform best among the four obesity-related indicators (TMI, WC z-score, BMI z-score, and BMI) to predict IR (31, 32).

Overall, TMI did not seem to be superior to BMI for predicting IR in children and adolescents. However, WHtR or WC could be a useful indicator for identifying IR among children and adolescents with overweight and obesity.

##### Blood Pressure

Only five studies compared the correlation of TMI and BMI with BP, with inconsistent results (**Table 2**) (13, 17, 21, 33, 43). Although BMI correlated with BP levels stronger than TMI (17, 33), one study based on 5,814 children and adolescents aged 6–19 years showed that, similar to BMI using a continuous variable, TMI (used as a continuous variable) had a low ability to identify high BP, with an AUC of only 0.66 to predict systolic BP and 0.60 to predict diastolic BP (21); similar findings were found among 57,201 Chinese children and adolescents aged 7–18 years, among 10,441 American adolescents aged 12–18 years (13) and among Italian adolescents (43).

Overall, only a few studies examined the question and they tended to suggest that either TMI or BMI performed poorly in identifying high BP in children and adolescents, and the ability varied in different populations.

##### Dyslipidemia

As shown in **Table 2**, three articles showed that both TMI and BMI poorly predicted dyslipidemia (13, 14, 21). Although using the same BMI classification, total cholesterol (TC) in boys and HDL-C and TG in girls were worse among children with overweight defined by TMI than among those with normal TMI (26), BMI (as a continuous variable) seemed to be more strongly associated with TC (R^2^: 0.32 *vs*. 0.27), HDL (-0.52 *vs*. -0.46), and TG (0.32 *vs*. 0.27) compared to TMI, while TMI (as a continuous variable) was more strongly associated with low-density lipoprotein-cholesterol (LDL-C) than BMI (0.38 *vs*. 0.33) (29), similar to findings on low HDL-C reported by Leone et al. (17), but inversely to findings by Alfaraidi et al. (42).

Overall, there are limited studies on the association of TMI and BMI with dyslipidemia components, and findings suggest that BMI performs better than TMI to identify high TC and TG, whereas TMI is superior to BMI to identify high LDL-C. This will need further evaluation.

##### Inflammatory and Liver Function Markers

As shown in **Table 2**, for C-reactive protein (CRP), the prediction accuracy of TMI and BMI z-score was similar (AUC: 0.74 *vs*. 0.73) (21), whereas other inflammatory markers including leptin, IL-6, and fetuin-A were more closely correlated with BMI than TMI (29). For liver enzymes, overweight and obese status based on TMI could significantly predict elevated serum glutamic oxaloacetic transaminase or elevated serum glutamic pyruvic transaminase, compared with overweight and obesity status based on BMI. However, different cutoffs were defined for BMI *vs*. TMI, which limits direct comparison (14). For non-alcoholic fatty liver, the discriminating ability of TMI was similarly poor as BMI, with AUC values of only 0.58 (44).

Overall, there is only limited evidence about the performance of TMI and BMI to predict inflammatory markers, which needs further research.

#### TMI in Childhood or Adolescence for Prediction of Specific CVRFs in Adulthood

Only four articles focused on the association of TMI *vs*. other obesity-related indicators in childhood or adolescence with CVRFs in adulthood (35–37, 46) (**Table 2**). BMI at ages 2 to 20 years predicted the presence of CVRFs in young adults aged 20 years as well or better than TMI. For example, the ability to predict adult IR, elevated insulin levels, and hypertension seemed to be stronger for BMI *vs*. TMI (as assessed in childhood), but similar for the prediction in adults of impaired fasting plasma glucose (FPG), low HDL-C, high LDL-C, high TG, and high aortic intima-media thickness (37). Similarly, another study showed that the AUC values for TMI, or for combination of TMI and BMI, did not outperform BMI alone in predicting adult obesity, diabetes, high carotid intima-media thickness, high LDL-C, and hypertension (35). The AUCs were low for TMI (0.53, 0.56, and 0.54), as well as for other adiposity indicators such as abdominal volume index (0.61, 0.62, and 0.61), BMI (0.59, 0.60, and 0.59), and WC (0.61, 0.61, and 0.61) in childhood to predict adult homeostasis model assessment 2-insulin resistance (HOMA2-IR), HOMA2-β, and high fasting insulin (36). However, when considering growth trajectory instead of a single measurement in childhood, a persistently high TMI during adolescence had predicted diabetes quite well in adults (AUC value as high as 0.81) (46).

Overall, TMI in childhood or adolescence seems to have a lower ability than BMI and other adiposity indicators to predict specific CVRFs in adulthood, whereas TMI trajectory has a higher ability than BMI trajectory in predicting diabetes in adulthood.

## Discussion

### Main findings

To the best of our knowledge, this is the first review to summarize the evidence regarding TMI as a screening tool for body fat and CVRFs in childhood and adulthood. TMI seemed to perform similarly or better than BMI for predicting body fat and central fat and performed similarly well as BMI in identifying MetS. However, the available evidence on the comparison of TMI and BMI (measured in childhood) for identifying specific CVRFs (in childhood or later in adulthood) including IR, high BP, dyslipidemia, and inflammation was limited and not compelling.

### TMI Performed Better Than BMI to Estimate Body Fat in Children and Adolescents

Unlike for adults, no standard BF% cutoff was established to define excess adiposity among children and adolescents until now (47), and objective measurements of fat mass [e.g., DEXA, doubly-labeled water (48), and isotope dilution technique (49)] were much complex and expensive. The components of TMI or BMI (weight and height) can be simply measured using the weight scale and the stadiometer that are widely used for routine pediatric clinical practice. Therefore, in this review, we compared the performance of TMI and BMI and our study suggested that TMI performed better than BMI to estimate body fat in children and adolescents at clinical practice.

The disadvantage of BMI and the advantage of TMI to estimate body fat are as follows. First, although BMI z-score seemed to predict well total fat mass, it predicted BF% weakly with altered body composition among adolescents (50). Second, the definition of overweight and obesity using BMI should be based on sex- and age-specific percentile values in childhood, but this requires using complex tables (10), which may overestimate the actual prevalence of adiposity in children, excessively worrying families and patients (50–53), particularly for adolescents who may be more prone to fat-shaming and weight bias (54). Third, TMI (which is defined independently of age and sex) could be simpler to use compared to age- and sex-stratified BMI cutoffs and a specific cutoff of TMI has been proposed (10). A better relation of TMI with body fat mass across age may be consistent with the fact that BF% may change largely during adolescence (possibly more among girls) due to the height spurt in this age range (55). Fourth, compared to BMI, TMI was more correlated with WHtR, which is a reliable clinical measure of abdominal obesity and is consistently associated with CVRFs (56). TMI could therefore help identify children and adolescents who are overweight or obese based on BMI but also have central obesity and increased risk of CVRFs.

### TMI Was More Simple and Accurate Than Other Indicators to Estimate Body Fat in Children and Adolescents

Although the RFMp calculated based on WC and height performed better to estimate BF% than TMI (25), the inter-operator variability between WC measurements is significant, which may cause more misclassification of MetS (57). Furthermore, for tall and thin people, WHtR may be unusually high, causing RFMp and RFM to tend to be 0 or negative (25). Therefore, considering the accuracy and simplicity of the use of TMI in primary health care services and its constancy in predicting adiposity at adolescence, TMI may be useful to evaluate body fat in adolescents. Yet, definite answers about the performance of BMI *vs*. TMI to predict adiposity in chidden and adolescents needs further studies using objective measurement of body fat mass (e.g., DEXA, isotope dilution) as the gold standard for comparisons, and do so in several populations, and within different ethnic, age, and sex groups.

### TMI Performed Similarly as Compared to BMI and Other Indicators in Predicting MetS and Its Components

Although TMI was superior to BMI to screen central fat (19, 24), in this review, it was similar to or not better than BMI to identify MetS and specific CVRFs. One possible reason might be that adiposity defined according to TMI or BMI only accounts for one of the MetS criteria. Another reason might be the inconsistent performances of three indicators (TMI, BMI, and WC) in identifying specific CVRFs including IR, high BP, dyslipidemia, and inflammation (13, 14, 21, 29, 31, 32), which are the main components of MetS.

### Age and Trajectory Influence the Association Between TMI *vs*. BMI in Childhood or Adolescence and Obesity-Related Morbidity in Adulthood

Although BMI in childhood or adolescence seemed to perform marginally better than TMI to predict obesity-related morbidity in adulthood (35, 36), the difference disappeared after adjusting for age (35), suggesting that age might be an important confounding factor that influences the association between BMI in childhood and obesity-related morbidity in adulthood. BMI was better than TMI only in late adolescence to predict adult IR and hypertension, suggesting that the variation of BMI during adolescence influences the strength of the association (10, 37). When considering trajectories, persistently high TMI during 13 and 18 years performed better than the BMI trajectory to predict adult diabetes (46), suggesting that, during adolescence, TMI trajectory (i.e., repeated measurements) may better reflect growth and predict adult CVRD outcomes. Therefore, further prospective studies with large sample sizes, multiethnic populations, and repeated measurements of anthropometric indicators are needed to confirm these findings.

### Strengths and Limitations

To the best of our knowledge, this is the first comprehensive review that compared TMI with BMI or other indicators in children and adolescents to predict obesity-related morbidity in both childhood and adulthood. Several limitations should be noted in this review. First, there was high heterogeneity between studies in the considered variables and how the adiposity cutoffs were defined, which limits direct comparisons. Second, most studies on the identification of CVRFs in childhood and adolescence were cross-sectional, which cannot prove causality (55). It must be however mentioned that a marker does not necessarily need to be causally related to an outcome to enable a good prediction. Third, a majority of the included studies came from Western countries, which limits the extrapolation of the results to other populations. Further studies with various ethnic/race groups are needed to confirm the predictive ability of TMI to predict adiposity in children and adolescents. Fourth, although TMI seems better than BMI to predict concomitant fat mass in children and adolescents, neither TMI nor BMI can distinguish fat mass from non-fat mass, and these indicators cannot replace objective measurement of fat mass (e.g., DEXA, isotope dilution). Again, an ultimate fully valid method to compare how BMI or TMI predicts adiposity should rely on objectively measured adiposity as the gold standard (e.g., DEXA, isotope dilution methods) and use a similar dichotomization of categories of elevated BMI or elevated TMI (e.g., using the same percentile cutoffs, e.g., p80 or p90) to enable valid comparisons; this was only rarely performed in the considered studies.

## Conclusions

In conclusion, TMI only requires a single threshold according to sex (i.e., no need for sex- and age-specific thresholds) and TMI seems to predict adiposity similarly or better in children and adolescents than BMI. In addition, TMI seemed to perform similarly as BMI for identifying MetS. However, the clinical use of TMI *vs*. BMI in childhood, in order to predict specific elevated CVRFs in childhood or later in adulthood, is still not definitive and needs further studies, particularly those with a longitudinal design.

## Data Availability Statement

The original contributions presented in the study are included in the article/supplementary material. Further inquiries can be directed to the corresponding author.

## Author Contributions

BX and PB designed the research. JS and RY conducted the literature search and performed the statistical analysis of the data. JS, BX, and PB wrote the manuscript draft. JS, BX, MZ, and PB contributed to the critical revision of the manuscript for important intellectual content. All authors contributed to the article and approved the submitted version.

## Funding

This work was supported by National Natural Science Foundation of China, Grant/Award Number: 81673195; Youth Team of Humanistic and Social Science of Shandong University.

## Conflict of Interest

The authors declare that the research was conducted in the absence of any commercial or financial relationships that could be construed as a potential conflict of interest.

## Publisher’s Note

All claims expressed in this article are solely those of the authors and do not necessarily represent those of their affiliated organizations, or those of the publisher, the editors and the reviewers. Any product that may be evaluated in this article, or claim that may be made by its manufacturer, is not guaranteed or endorsed by the publisher.
